# Mechanical Response and Failure Mechanisms of Natural Bamboo Fiber Reinforced Poly-Benzoxazine Composite Subjected to Split-Hopkinson Tensile Bar Loading

**DOI:** 10.3390/polym14071450

**Published:** 2022-04-02

**Authors:** Kai Zhang, Fangxin Wang, Bin Yang, Lin Li, Li Gao, Yongyang Sun, Fuzheng Guo

**Affiliations:** 1 School of Civil Engineering and Architecture, Suqian University, Suqian 223800, China; ll86268555@163.com (L.L.); gaoli@squ.edu.cn (L.G.); 2 College of Aerospace and Civil Engineering, Harbin Engineering University, Harbin 150001, China; sunyongyang@hrbeu.edu.cn; 3 Department of Mechanical Engineering, National University of Singapore, Singapore 117576, Singapore; 4 School of Civil Engineering and Architecture, Yangzhou University, Yangzhou 225000, China; wangfangxin@yzu.edu.cn (F.W.); mz120211036@yzu.edu.cn (F.G.); 5 School of Aerospace Engineering and Applied Mechanics, Tongji University, Shanghai 200092, China; yangbin_tj@tongji.edu.cn

**Keywords:** bamboo fiber, benzoxazine matrix, strain rates, tensile properties, failure mechanisms

## Abstract

In this study, chopped natural bamboo fibers were successfully added in the benzoxazine matrix by the hot-pressing method to fabricate environmentally friendly bio-composite. The mechanical behaviors and failure mechanisms of neat benzoxazine matrix and its bamboo fiber composite under different tensile strain rates (quasi-static, 35/s and 110/s) were comparatively investigated using SHTB device (split-Hopkinson tensile bar), high-speed camera, DIC method (digital image correlation), and SEM observation (scanning electron microscopy). The results showed the composite exhibited 30.02% and 25.21% higher strength than that of neat benzoxazine under strain rates of 35/s and 110/s, respectively. However, under quasi-static tensile loading, the tensile strength of the composite was not higher than that of neat benzoxazine. The SEM and high-speed camera images showed the bamboo fibers displayed different reinforcing mechanisms under different strain rates. The chopped bamboo fibers could strengthen the composite effectively under dynamic tensile loadings. However, under quasi-static loading, the tensile strength of the composite was largely determined by the potential defects (such as small bubbles, pores, and fiber agglomerations) in the composite.

## 1. Introduction

In order to deal with the challenges of energy shortage and environmental pollution, various types of natural fibers have attracted extensive attention from the scientific and business fields in recent decades [1,2,3]. Compared with petroleum-based chemical fibers (carbon, aramid, glass, etc.), natural fibers have many excellent characteristics, such as low cost, abundantly available, renewability, light weight, and high strength, thus demonstrating great potential in fabricating fiber-reinforced polymer composites [4,5,6]. Natural fibers, especially plant fibers, such as coir, cotton, flax, hemp, jute, bamboo, etc., have been successfully applied in manufacturing bio-composites [7,8,9]. However, some inherent defects of natural fibers (limited stiffness and strength, complicated preparation process, strong hydrophilicity, etc.) restrict their further development and application in high-level industries [4,10]. Moreover, according to Chinese market research, more than 90% of fiber composites still use glass fiber as reinforcing material currently. This result indicates that more fundamental research needs to be carried out to look for strategies and breakthroughs in producing high-performance natural fibers and composites.

At present, substantial research focuses on surface pre-treatment, water absorption behavior, quasi-static mechanical properties, thermal properties, and preparation methods of natural fiber-reinforced composites [11,12,13,14]. Alkaline, silane, maleated, and acetylation treatments were commonly used to address the incompatibility and poor adhesion between natural fibers and hydrophobic matrices [11]. These surface modification methods were proved to be useful for improving the mechanical properties of natural fiber reinforced composites to some extents. However, hybridization of natural fibers with chemical fibers, such as carbon, glass, and aramid fibers, was deemed as a more effective way to significantly enhance the mechanical properties of resulted composites [15,16]. Another technique barrier to further application of natural fibers is the serious degradation of mechanical properties of its composites after long-term water immersion. Water molecules could cause holes and micro-cracks in composites, weaken the interfacial bonding between natural fibers and polymer matrices, and hence, decrease the stiffness and strength of the composites [12]. Due to the relatively lower mechanical properties, natural fibers were mainly used as fillers for producing some semi-structural and non-structural composites and the majority of the published literature was limited to the routine quasi-static mechanical experiments of the corresponding composites [4].

However, with the gradual deterioration of the global environment, the governments all over the world have put forward the carbon neutralization strategy, the development and utilization of natural fiber is an irresistible trend. In addition, natural fibers could become a potential substitute for synthetic fibers in many sectors, such as aerospace, automotive, marine, buildings, leisure goods, electronic appliances, handphone casing, biomedical, and military [3]. Meanwhile, natural fiber composites still face the challenges and threats of high-temperature environments, dynamic loadings, hydrothermal aging, etc. For example, some automobile door panels made of natural fiber composites face the risk of impact loadings in practical service. However, only few studies focused on the mechanical behaviors and failure mechanisms of natural fiber composites under impact loading conditions. Especially, to the best of our knowledge, there are no published studies on the chopped bamboo fiber-reinforced poly-benzoxazine composite subject to SHTB loading.

In this paper, a novel polymer material-benzoxazine resin and bamboo fibers were selected as matrix and reinforcement, respectively. As a high-performance thermosetting resin, benzoxazine exhibits many superior properties, such as environmentally-friendly, high glass transition temperatures, no shrink during curing, flame retardant, low water uptake, strong designability, etc. [17,18,19]. Benzoxazine resin demonstrated great potential in the polymer engineering field in the future. Bamboo plants, as wood materials for poor countries are widely distributed in Asia, Africa, South America, and other regions [20]. Bamboo plants have a strong CO_2_ sequestration capacity and are becoming a popular bio-material. The natural bamboo fibers are extracted from mature bamboo directly and exhibit excellent mechanical and other functional properties [21,22]. More and more research focuses on the preparation and properties of bamboo fiber/polymer composites.

Plant fibers exhibit a high strength–weight ratio and are deemed as a potential alternative for synthetic fibers. The composites made of plant fibers will inevitably face the threat of impact loading when the composites are used in automobiles, aircraft, and other fields. However, very few studies investigate the mechanical properties and failure mechanisms of the plant fiber composites subjected to dynamic loadings with different strain rates. Therefore, in our study, chopped bamboo fibers were successfully added to the benzoxazine matrix by the hot-pressing method. The mechanical responses and failure mechanisms of neat benzoxazine and bamboo fiber-reinforced poly-benzoxazine composite were comparatively investigated by SHTB system, high-speed camera, DIC method (digital image correlation), and SEM observation (scanning electron microscopy). Many details on how the chopped bamboo fibers work when the composite was stretched at different strain rates were discussed. This paper could provide some useful information to expand the utilization of such kind of composite in the field of impact engineering.

## 2. Experimental Section

### 2.1. Materials

In this study, a novel thermosetting resin-benzoxazine was used as a polymer matrix. The type of benzoxazine resin was diaminodiphenylmethan-benzoxaizne (commercial name was high-temperature resistance benzoxazine, Coryes Polymer Science & Technology Co., Ltd., Chengdu, China). The mechanical parameters of bamboo fibers are determined by various factors, such as the type of bamboo plants, age, growth environment, etc. [23]. In our study, natural bamboo fibers extracted from Zhejiang Province wasneosinocalamus affinis (commercial name was Single bamboo fiber) were kindly supported by ShengZhu Co., Ltd. (Ningbo, China). The density and average diameter of bamboo fibers were 0.72 g/cm^3^ and 136 µm, respectively. The lengths of the bamboo fibers were sized into 5 mm manually. The bamboo fibers were composed of about 78.52% cellulose, 6.75% hemicellulose, 10.14% lignin, and some other colloidal substances. The mechanical properties of the selected bamboo fibers could be easily found in our previous work [14]. In addition, based on our previous study, 6 wt.% NaOH solution was used to pre-treat the bamboo fibers for removing the hydrophilic materials on the fiber surface.

### 2.2. Composite Preparation

**Figure 1:**
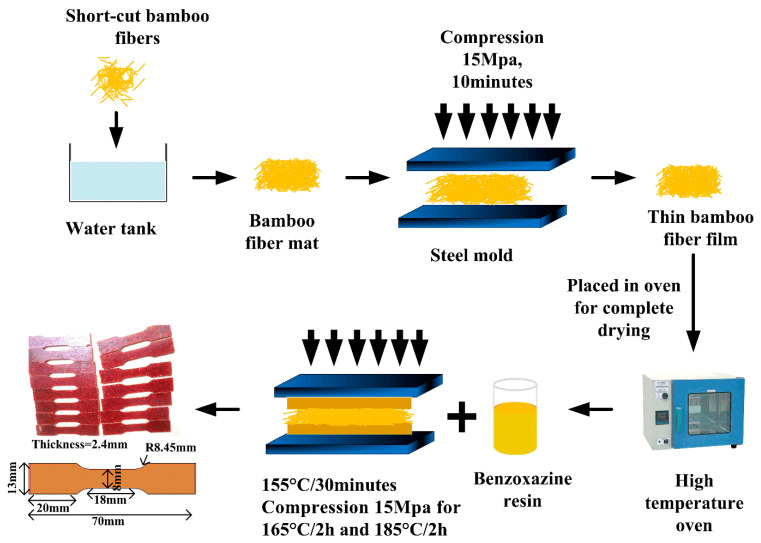
Schematic diagram of preparation of the chopped bamboo fiber-reinforced poly-benzoxazine composite.

Unlike synthetic fibers, chopped bamboo fibers are very fluffy, and it is very difficult to add high content fibers into the benzoxazine matrix uniformly. Therefore, in this paper, we developed a simple and low-cost method to prepare bamboo fiber mat and fabricated chopped bamboo fiber reinforced poly-benzoxazine composite. Firstly, as shown in Figure 1, pre-treated bamboo fibers (length ~5 mm) were poured into a water box and stirred carefully. The colloidal layer on the surface of bamboo fibers could dissolve and transfer in water, so that the chopped bamboo fibers could adhere to each other to form a fiber mat. Secondly, the water was squeezed out of the water box with a plastic plate and the bamboo fiber mat was then placed on the plate. The wet and fluffy bamboo fiber mat needed to be turned into a completely dry and thin mat using an oven and hot-press machine, respectively. The dried and thin bamboo fiber mat was then carefully stored in glass bags. Benzoxazine resin would become liquid state at 120–160 °C and cure at temperatures beyond 160 °C. Therefore, thirdly, benzoxazine resin was heated in the oven and half of melted benzoxazine resin was poured into the steel mold and then the thin and dry bamboo fiber mat was carefully placed on the resin. The rest of the melted resin was further poured uniformly on the surface of the fiber mat. The temperature of the hot-pressing machine was set to 155 °C for increasing the viscosity of the fiber/resin mixture. Lastly, the upper steel mold was closed and compressed at 165 °C and 185 °C for 2 h (the pressure was set as 15 MPa), respectively. To prevent deformation of the composite, take out the composite until the temperature drops to about 25 °C. The specimens were sized by a water cutting machine and the dimensions are shown in Figure 1. The weight fraction of bamboo fibers was 20 wt.%, and the neat benzoxazine was also prepared for comparison.

### 2.3. Quasi-Static Tensile Test

**Figure 2:**
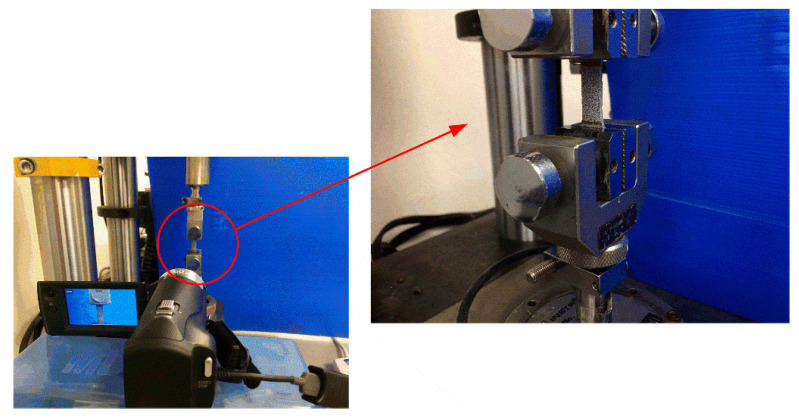
Quasi-static tensile test based on universal Instron testing machine.

As shown in Figure 2, the quasi-static tensile properties of neat benzoxazine and its bamboo fiber composite were tested by a universal Instron machine (Instron 3400, 10 kN, Instron^®^ GmbH, Darmstadt, Germany). The test was carried out at room temperature and the speed of the fixture was 1 mm/min. To eliminate size effects, the dimensions of specimen subjected to dynamic loadings were the same as the quasi-static ones. The deformation process of the specimen was recorded with an ordinary camera. For better accuracy, the strain of the specimen was determined by spraying speckles on the surface of the specimen and a commercial DIC (digital image correlation, MatchID-2D/Stereo, LTY Co., Ltd., Beijing, China) software.

### 2.4. Impact Tests

**Figure 3:**
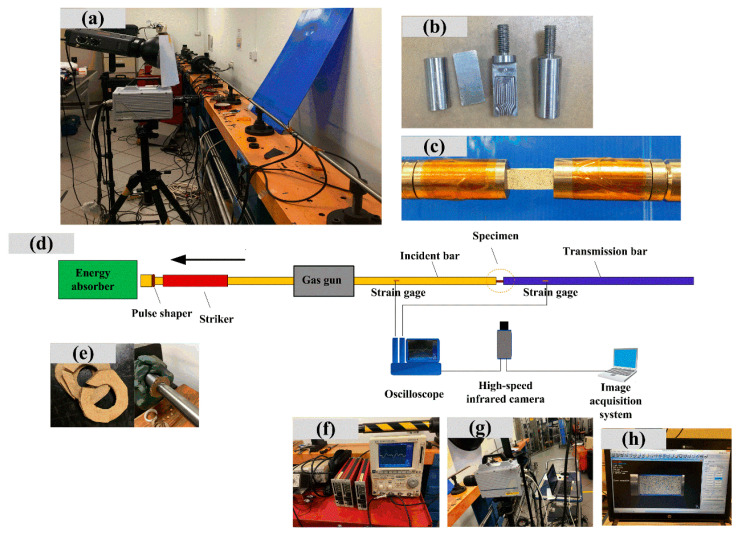
Split Hopkinson tensile bar system with a high-speed camera. (**a**) SHTB equipment, (**b**) Self-designed fixture, (**c**) Specimen with black and white speckles, (**d**) Schematic diagram of SHTB system, (**e**) Cardboard pulse-shapers, (**f**) Oscilloscope and strain instrument, (**g**) High-speed camera, (**h**) Data acquisition.

As shown in Figure 3, the split Hopkinson tensile bar testing system was commonly used in experimental research of polymer composites subjected to different strain rates [24,25,26,27]. Figure 3a–h shows SHTB system, fixture of specimen, testing sample with black and white speckle, schematic diagram of testing system, cardboard pulse shaper, oscilloscope, dynamic strain gauges, high-speed camera, and image acquisition machine, respectively. The lengths of the striker, incident bar, and transmitted bar are 500, 3000, and 2000 mm, respectively. The diameter of the bars is 16 mm, and the material of the bars is titanium alloy with the Young′s modulus *E* = 110 GPa and density *ρ* = 4.4 g/cm^3^. In the impact test, when the striker hits the end of the incident bar, a tensile wave is generated. The high-speed camera starts to work when the input signal is monitored by a strain gauge and oscilloscope. It should be pointed out that because the tensile strength and fracture strain of the poly-benzoxazine composite are very low, the traditional foil resistance strain gauge is not enough to accurately measure the strain on the transmission bar. Therefore, a high-precision semiconductor strain gauge (KSPB-3-120-F2-11, 120 Ω, KYOWA Co., Ltd., Osaka, Japan) was used in this study. The stress and strain were calculated by the one-dimensional elastic wave theory. The engineering strain ($\varepsilon_{E}\;\left(t\right)$) and engineering stress ($\sigma_{E}\;\left(t\right)$) of the sample were determined by the following equations [24]:

(1) $$\varepsilon_{E}=-\frac{2C_{0}}{L_{s}}\int_{0}^{t}\varepsilon_{R}\left(t\right)dt$$

(2) $$\sigma_{E}=\;\frac{A_{0}}{A_{s}}\varepsilon_{T}\left(t\right)$$

In the equations, $E_{0}$, $A_{0}$, and $C_{0}$ refer to Young’s modulus, the cross-sectional area of the incident bar, stress wave speed, respectively. $L_{s}\;$ and $A_{s}$ are the length and cross-sectional area of the specimen, respectively. $\varepsilon_{R}\;\left(t\right)$ is the tensile strain history of the incident bar and $\varepsilon_{T}\;\left(t\right)$ is the tensile strain history of the transmitted bar tested by the applied high-precision semiconductor strain gauge. The average strain rate of the testing sample was simply determined by high-speed camera and DIC technique. To ensure the reliability of the experiment, two repeated experimental results of same specimen were needed to be obtained at least.

### 2.5. SEM Observation

Scanning electron microscopy (SEM, Gemini 500, Carl Zeiss Jena, Oberkochen, Germany) was carried out to analyze the effects of different strain rates on the failure modes of bamboo fiber composite under SHTB loadings. SEM test was conducted in the high vacuum mode at accelerating voltages of 15 kV.

## 3. Results and Discussion

### 3.1. Tensile Behaviors of the Neat Benzoxazine and Its Composite under Quasi-Static Loading

**Figure 4:**
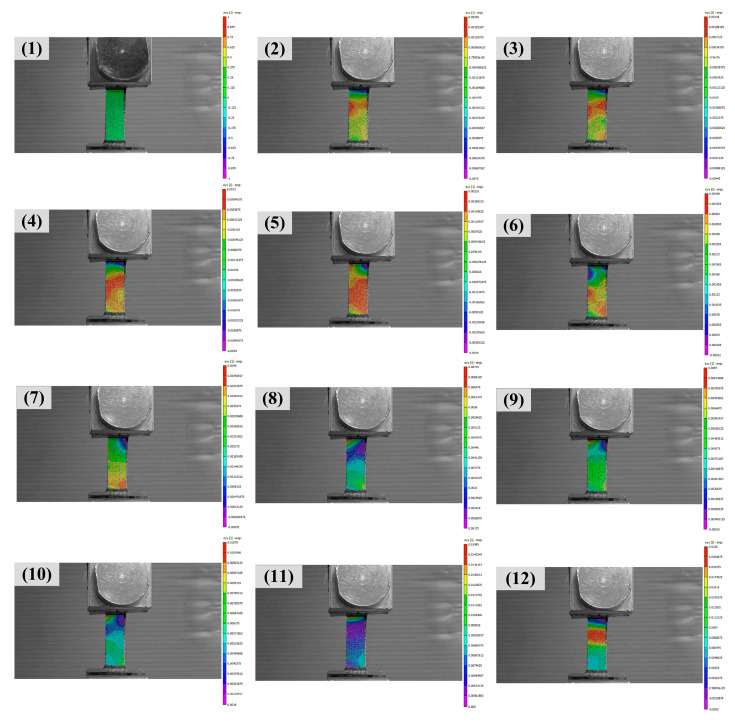
The strain history of specimen under quasi-static loading determined by DIC method. (**1**)–(**12**) the whole deformation process of the specimen.

In quasi-static experiment, the stress of the testing sample was obtained directly from the Instron machine. Because the fracture strain of neat benzoxazine was extremely low, the strain was determined by professional DIC software for better accuracy. As shown in Figure 4, the whole deformation process could be recorded by spraying black-and-white speckles on the sample. The testing sample broke near the middle (as shown in Figure 4 (12)), and this meant the obtained data were reliable.

**Figure 5:**
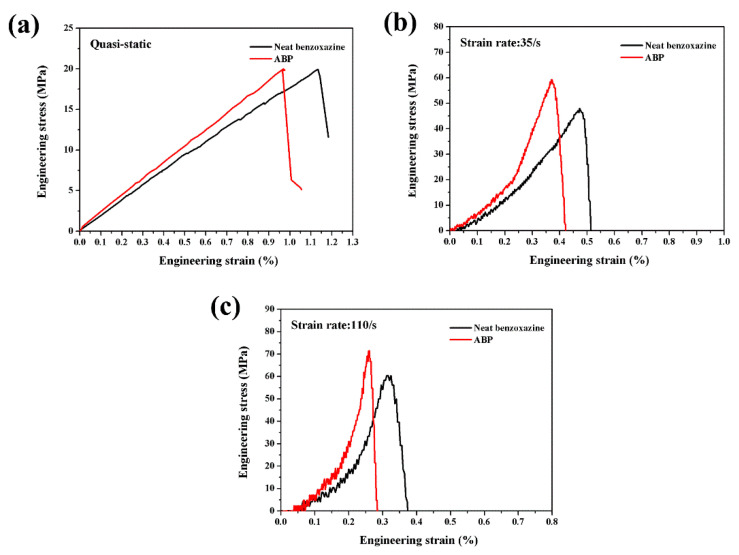
The tensile stress-strain curves of neat benzoxazine and its composite under different strain rates. (**a**) Quasi-static, (**b**) 35/s, (**c**) 110/s.

Figure 5a–c presented the typical tensile stress-strain curves of neat benzoxazine and ABP (Alkali-treated 20 wt.% bamboo fiber-reinforced poly-benzoxazine composite) under quasi-static loading, strain rate of 35/s and 110/s, respectively. Obviously, both the neat benzoxazine matrix and ABP exhibited complete linear elastic characteristics under quasi-static loading conditions. This result was similar to some relevant research [28,29]. In this paper, the quasi-static test showed that chopped bamboo fibers had very little effect on the tensile strength of the composite. 

However, Dayo et al. [28] studied the tensile properties of chopped hemp fiber-reinforced poly-benzoxazine composites and the results indicated short-cut hemp fibers could enhance the tensile strength of the composite. In our study, the high strength of bamboo fiber could not be used effectively because these fibers were uniformly dispersed rather than added in a unidirectional way [30]. More importantly, potential defects, such as small bubbles, pores, and fiber agglomerations, which were generated during the preparation process, could greatly weaken the strength of the composite. Under quasi-static loading condition, the testing specimen could fail firstly at the defective areas. In other words, the strength of the composite was mainly controlled by its internal defects. Manalo et al. [31] studied the mechanical properties of bamboo fiber-polyester composites and found that chopped bamboo fibers could only be used as fillers rather than reinforcement in the composite. Table 1 presents the ultimate stress and strain of neat benzoxazine and its composite under different strain rates. The fracture strains of neat benzoxazine and ABP were only 1.16% and 0.94%, respectively. Compared with neat benzoxazine, pure epoxy-the most commonly used thermosetting resin, had obvious higher strain at break [32]. Unfortunately, the fracture strain of neat benzoxazine was further reduced by adding chopped bamboo fibers to the matrix.

### 3.2. Tensile Behaviors of the Neat Benzoxazine and Its Composite under SHTB Loading

**Figure 6:**
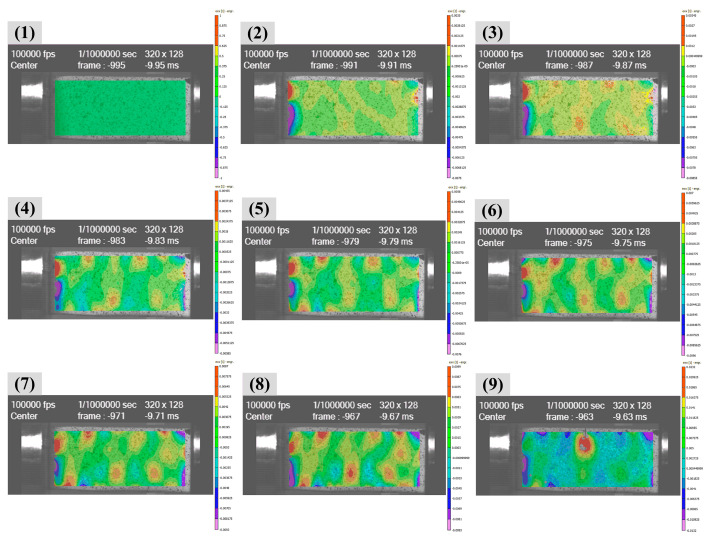
The strain of specimen under SHTB loading was determined by the high-speed camera DIC method. (**1**)–(**9**), the whole deformation process of the specimen.

Similarly, the strain of the specimen under SHTP loadings was also calculated by a high-speed camera and the same DIC software. For the reliability of the experiment, more than two repeated experimental data need to be gotten for each specimen. As shown in Figure 6 (9), we recorded the experiment data of the testing sample, which broke near the middle. 

**Figure 7:**
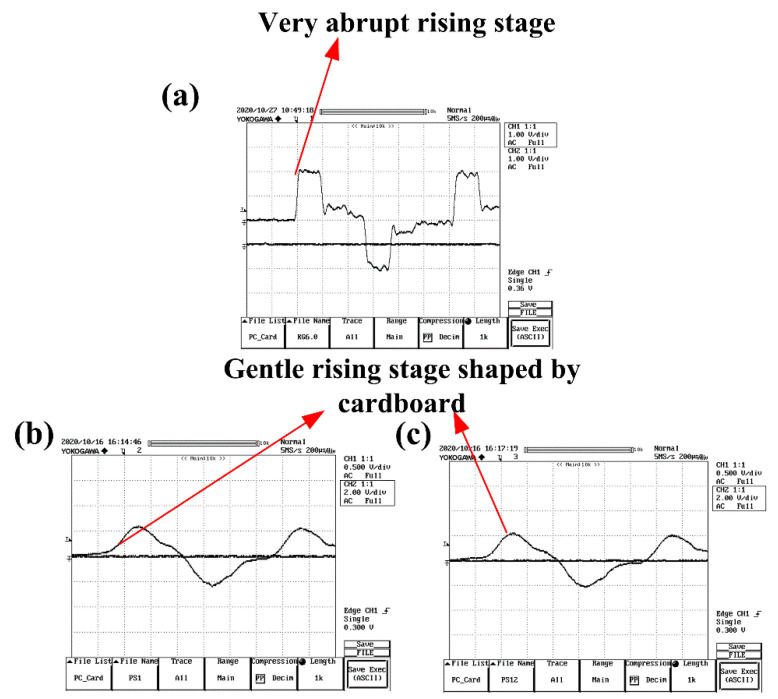
The incident stress wave patterns with and without cardboard as pulse shaper. (**a**) Very abrupt rising stage without cardboard, (**b**) Gentle rising stage shaped by cardboard, (**c**) Cardboard exhibited good consistent.

Based on the experimental results under quasi-static loading conditions, both neat benzoxazine and the composite were very brittle. The tensile strain and strength of APB were only 0.94 and 20.6 MPa, respectively. Therefore, the stress equilibrium of the SHTB testing specimens was very important and necessary for valid experimental results under dynamic loadings. Therefore, as shown in Figure 7, we selected cardboards as pulse shaper in our study. As shown in Figure 7a, without a pulse shaper, the incident wave displayed a typical square pattern. Comparatively, as shown in Figure 7b,c, the carboards could perfectly shape the incident wave into a trigonometric function pattern. Moreover, the cardboards as pulse shapers showed good consistency. With pulse shapers, the specimens could have more time to reach the stress equilibrium state before failure. 

According to Refs [33,34], the stress equilibrium of the split Hopkinson bar test could be determined by the following equations:

(3) $$e_{k}=\frac{F_{s-i}^{k-1/2}F_{s-t}^{k-1/2}}{1+F_{s-t}+F_{s-i}F_{s-t}+F_{s-i}F_{s-t}{}^{2}+\ldots +F_{s-i}^{k-1/2}F_{s-t}^{k-1/2}}$$

(4) $$F_{s-t}=\frac{1-n_{s-t}}{1+n_{s-t}}$$

(5) $$n_{s-t}=\frac{{\left(\rho c_{0}A\right)}_{s}}{{\left(\rho c_{0}A\right)}_{t}}$$

In the above equations, $e_{k}$ refers to the non-equilibrium coefficient and ρ, *c*_0_, *A* represent density, stress wave speed, and cross-sectional area, respectively. Subscripts *k*, *s*, *i* and *t* mean reflection times of stress wave in the sample, sample, incident bar, and transmitted bar, respectively. The stress equilibrium of the testing sample could be guaranteed if the non-equilibrium coefficient ($e_{k}$) was less than 5%. In our study, the whole deformation process of the testing sample could be recorded by a high-speed camera and DIC technique. The stress equilibrium for each testing sample was checked before exporting experimental data.

The typical stress-strain curves of neat benzoxazine and the composite under SHTB loadings can be found in Figure 5b,c. Interestingly, under dynamic loadings, APB displayed obvious higher tensile strength and modulus than neat benzoxazine. This meant that chopped bamboo fibers could improve the tensile properties of the composite. This was an interesting result. As discussed above, the chopped bamboo fibers could not enhance the strength of the composite under quasi-static loading. However, under dynamic loading, the bamboo fibers displayed completely different reinforcing mechanisms. Similar results could be found in our previous study [35]. According to Ref. [35], the chopped bamboo fibers had a negative effect on the quasi-static compressive property of the composite. Under SHPB loadings, the bamboo fibers could obviously improve the strength and toughness of the composite. The results further proved that the internal defects in the composite could not significantly affect the mechanical properties of the composite subjected to dynamic loadings. During the SHTB test, the specimen would break very quickly, and there was not enough time for the defects (bubbles, pores, and fiber agglomerations in the composite) to develop. Therefore, the bamboo fibers could enhance the tensile strength of the composite. It should be noted that further studies are still needed to clarify the reinforcing mechanisms. 

Lastly, it was noted that the stress-strain curves of both neat benzoxazine and its composite did not display perfect linear elastic behavior. However, it was widely acknowledged that the Young′s modulus of polymer materials tested by the split-Hopkinson bar systems was not accurate [33]. Therefore, the nonlinear behavior was not considered as the mechanical property of the material itself and the values of Young′s modulus of the specimens were not given in this study.

### 3.3. Rate Dependence of Tensile Behavior of Neat Benzoxazine and Its Composite

**Figure 8:**
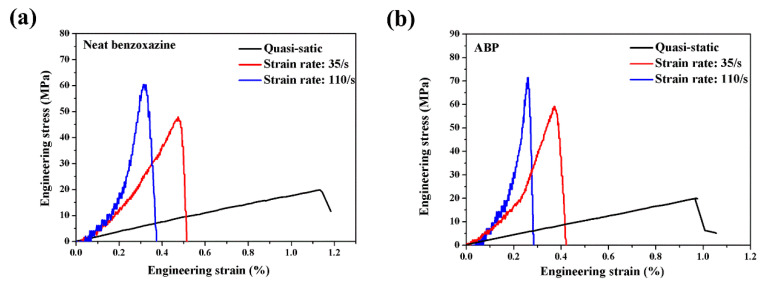
Rate dependence of tensile behavior of neat benzoxazine and its composite. (**a**) Neat benzoxazine, (**b**) ABP.

The effects of strain rates on the tensile properties of neat benzoxazine and its composite were presented in Figure 8. To the best of our knowledge, there was no published paper to discuss the strain rate dependence of such a novel thermosetting polymer material. As shown in Figure 8a, with the increase in strain rates, the benzoxazine matrix became more brittle, and the specimen broke at a very low strain value. The average fracture strains of neat benzoxazine at the strain rate of quasi-static, 35/s and 110/s were 1.16, 0.52 and 0.34, respectively. Unfortunately, ABP displayed more brittle properties at the same strain rates. However, the ultimate strengths of APB at the strain rate of 35/s and 110/s were 60.2 MPa and 74.5 MPa, respectively. In comparison, at the same strain rates, the strengths of neat benzoxazine were only 46.3 MPa and 59.5 MPa, respectively. As thermosetting resin, benzoxazine displayed obvious positive strain rate effects. This result was expected because many similar polymer materials had the same characteristics [36]. However, for bamboo fiber composite, the strain rate effects could be affected by both the benzoxazine matrix and the bamboo fibers. The strain rate sensitivity of bamboo fiber itself needs to be studied in the future. To conclude, benzoxazine resin had positive strain rate dependence, and this polymer material demonstrated great potential in the field of impact engineering.

### 3.4. Failure Mechanisms 

**Figure 9:**
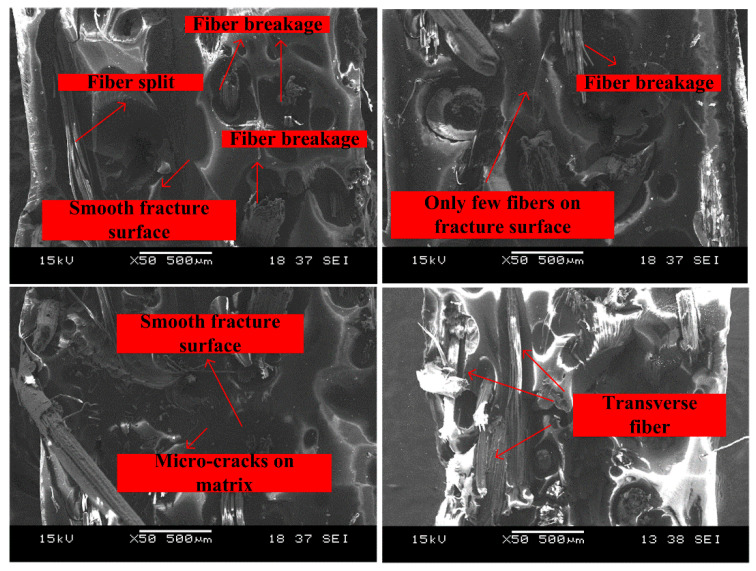
Fracture morphologies of APB under quasi-static conditions.

To further study the effects of strain rate on the failure mechanisms of the bamboo fiber reinforced poly-benzoxazine composites, SEM test was carried out in this paper. Figure 9 shows the representative SEM images of the fracture surface of APB under quasi-static conditions. From the images, it could be found that the fracture surface was relatively smooth, and only a few bamboo fibers were exposed on the surface. Moreover, most of the exposed bamboo fibers were transverse fibers. As we know, only the fibers along the direction of tensile loadings could enhance the modulus and strength of the composite [31]. Therefore, as shown in Figure 9, the exposed transverse bamboo fibers did not beak and had no positive effects on strengthening the composite. As discussed above, under quasi-static loading conditions, the strength of the composite was mainly dominated by the potential defects (such as small bubbles, pores, and fiber agglomerations) in composite. Chopped bamboo fibers could not be completely evenly dispersed in benzoxazine resin. This may lead to the failure of composite in the area where the content of bamboo fibers was relatively lower. This explains why only a few fibers appeared on the fracture surface and the composite did not show higher strength than that of neat benzoxazine. 

**Figure 10:**
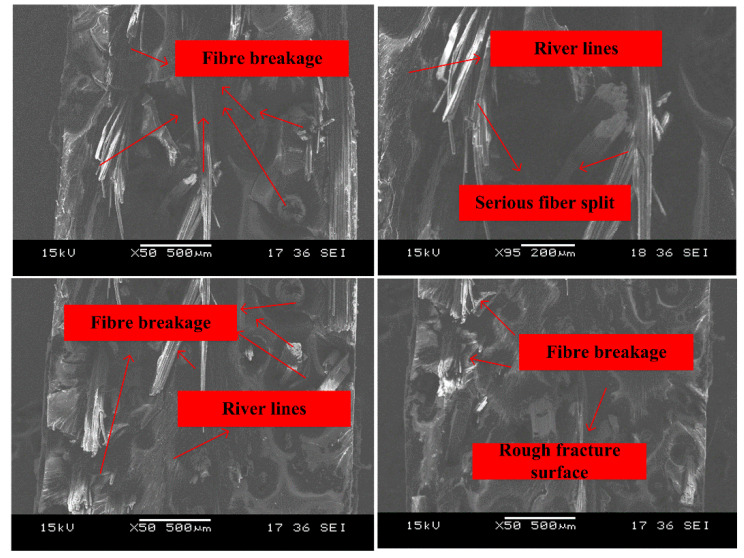
Fracture morphologies of APB under strain rate of 35/s.

Figure 10 shows the fracture surface of the composite subject to SHTB loading at the strain rate of 35/s. It was obvious that fiber breakage, fiber split, and river lines could be observed on the fracture surface. Compared with the specimen under quasi-static loading, much more broken bamboo fibers were exposed on the fracture surface. According to Ref. [14], the ultimate strength of bamboo fibers was almost 15 times that of neat benzoxazine. Under dynamic loadings, the bamboo fibers could effectively strengthen the composite. Moreover, few bamboo fibers were pulled out during the loading process. This indicated that the composite had strong interfacial bonding between the alkali-treated bamboo fibers and benzoxazine matrix. The river lines and the rough fracture surface could further prove the bamboo fibers contributed to the high tensile strength of the composite. The SEM images supported the experimental data of SHTB test. Under SHTB loading condition, the testing specimen broke instantly and the defects in the composite may had not enough time to propagate and hence the bamboo fibers could work effectively. 

**Figure 11:**
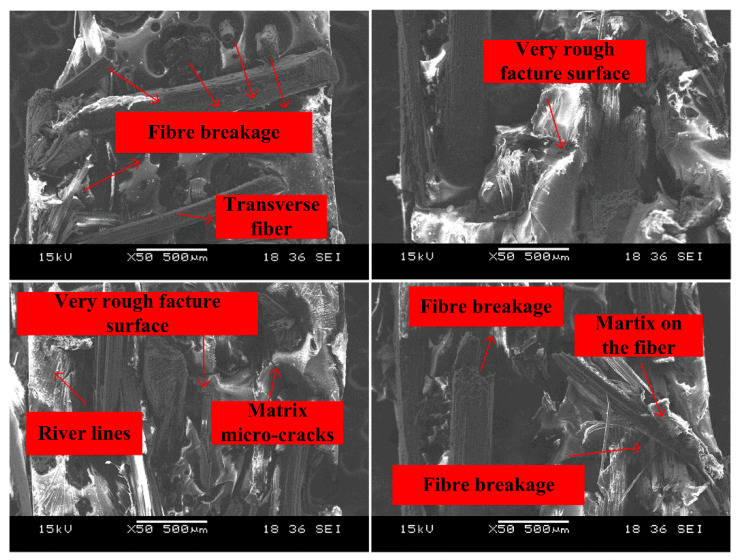
Fracture morphologies of APB under the strain rate of 110/s.

Under a higher strain rate of 110/s, Figure 11 shows the fracture surface of the specimen became rougher. Matrix cracking, fiber breakage, and split were the main failure modes. It was worth noting that some benzoxazine resin could be observed on the bamboo fibers and this further indicates the interfacial bonding between fibers and the matrix was still very strong at a higher strain rate.

**Figure 12:**
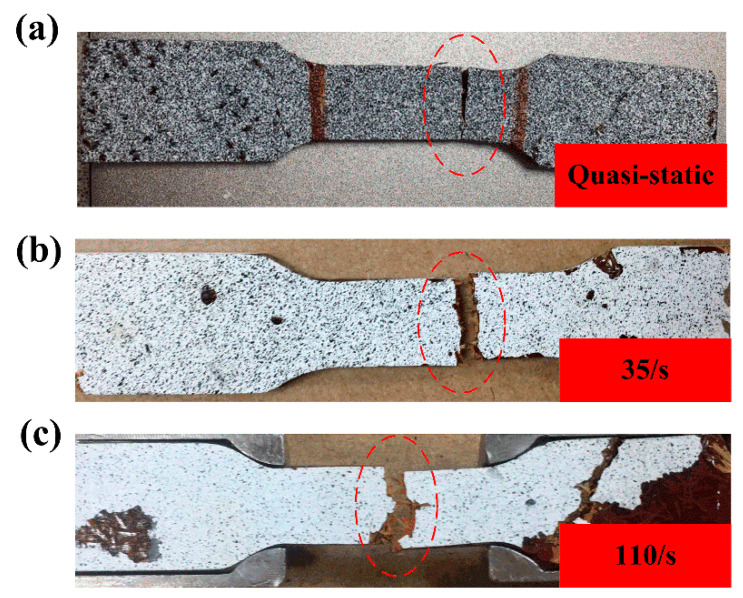
Fracture morphologies of the composite under different strain rates. (**a**) Quasi-static, (**b**) 35/s (**c**) 110/s.

**Figure 13:**
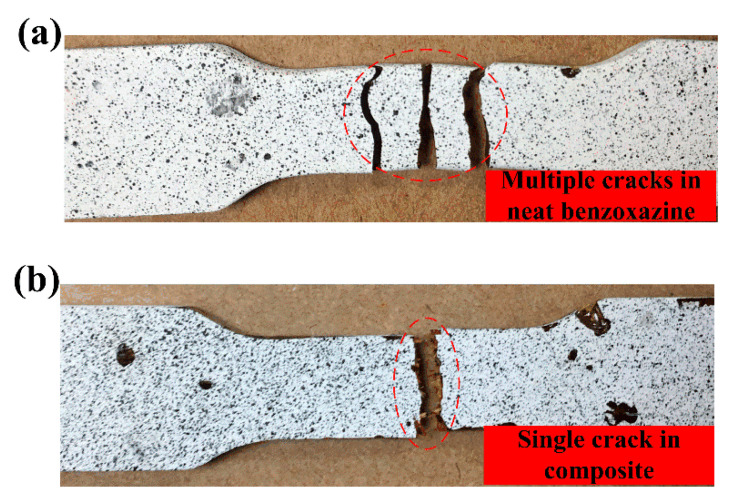
Bamboo fibers could effectively hinder the rapid propagation of cracks in composites. (**a**) Neat benzoxazine matrix, (**b**) ABP.

Figure 12a–c showed the fractured specimens under quasi-static loading conditions, the strain rate of 35/s and 110/s, respectively. The red circles marked the areas of fracture surfaces. Obviously, the fracture surface became more and more uneven and rugged with the increase in strain rates. This result was consistent with the SEM images. Compared with Figure 12a, much more exposed bamboo fibers could be observed in Figure 12c. Under SHTB loading conditions, a large number of microcracks propagated in the composite, and the existence of bamboo fibers would hinder the crack propagation to some extent. Therefore, the chopped bamboo fibers could improve the tensile strength of the composite under dynamic loadings. This guess could be proved by Figure 13. As shown in Figure 13, the neat benzoxazine broke with multiple cracks, and the specimen would easily break into several pieces under SHTB loadings. However, during the SHTB experiment, all the bamboo fiber/poly-benzoxazine composite failed with only one macro-crack. This further indicates the bamboo fibers could hinder the propagation of cracks effectively.

## 4. Conclusions

In this study, short-cut bamboo fibers were successfully added to the benzoxazine matrix by the traditional hot-pressing method, and neat benzoxazine was also prepared for comparison. The tensile behaviors of neat benzoxazine and its composite under various strain rates were comparatively studied by a universal Instron machine and SHTB system. With a high-speed camera, DIC method, and SEM observations, the failure mechanisms of the composite under different strain rates were carefully analyzed. The following are the main conclusions of this study:

1. The chopped bamboo fibers could not increase the tensile strength of the composite under quasi-static loading conditions. However, the results showed the composite exhibited 30.02% and 25.21% higher strength than that of neat benzoxazine under the strain rate of 35/s and 110/s, respectively.
2. The tensile behavior of the composite under quasi-static loading was mainly controlled by the potential defects (such as small bubbles, pores, and fiber agglomerations) in the composite.
3. Under SHTB loadings, the chopped bamboo fibers exhibited obvious positive effects on the dynamic tensile properties of the composite. The potential defects in the composite may have not enough time to develop due to the extremely high strain rates. The neat benzoxazine and its composite displayed a positive strain rate effect.
4. With the increase in strain rates, fiber breakage, fiber split, and river lines could be observed on the fracture surface of the composite. Compared with the specimen under quasi-static loading, much more broken bamboo fibers were exposed on the fracture surface. The existence of bamboo fibers could effectively hinder the rapid propagation of cracks in the composite.
5. The chopped bamboo fibers were proven to exhibit positive effects on the dynamic mechanical properties of the composite. This demonstrated plant fibers could be used in the impact engineering field. However, further research is needed for studying the strain rate effects of plant fibers.

## Figures and Tables

**Table 1:** Ultimate stress and strain of neat benzoxazine and ABP under different strain rates.

Strain Rate (s^−1^)	Ultimate Stress (MPa)/Strain (%)		
	Neat Benzoxazine		ABP
**0.001**	20.5 ± 1.3/1.16 ± 0.11		20.6 ± 1.1/0.94 ± 0.13
**35**	46.3 ± 3.2/0.52 ± 0.08		60.2 ± 5.8/0.41 ± 0.09
**110**	59.5 ± 3.3/0.34 ± 0.07		74.5 ± 4.5/0.24 ± 0.08

## Data Availability

The data is all in the article.
